# Unplanned Post‐Operative Pediatric Intensive Care Unit Admissions After Elective Upper Airway Procedures: A Retrospective Case–Control Study

**DOI:** 10.1002/pan.70185

**Published:** 2026-04-11

**Authors:** Hayes Stancliff, Michelle M. Basilious, Lisa R. Yoder, Conrad Krawiec, Terrence Murphy, Priti G. Dalal

**Affiliations:** ^1^ Department of Medical Education Pennsylvania State College of Medicine Hershey Pennsylvania USA; ^2^ Robert Wood Johnson University Hospital New Brunswick New Jersey USA; ^3^ University of Michigan Medical School Ann Arbor Michigan USA; ^4^ Department of Pediatrics Penn State Health Children's Hospital Hershey Pennsylvania USA; ^5^ Department of Public Health Sciences Penn State College of Medicine Hershey Pennsylvania USA; ^6^ Department of Anesthesiology Penn State Health Milton S. Hershey Medical Center and Pennsylvania State College of Medicine Hershey Pennsylvania USA

**Keywords:** intensive care units, obstructive sleep apnea, pediatric, pediatric anesthesia, postoperative complications, risk assessment

## Abstract

**Background:**

Elective pediatric upper airway procedures are generally safe; however, some patients require unplanned pediatric intensive care unit admission, increasing care complexity.

**Aims:**

To identify perioperative risk factors associated with unplanned pediatric intensive care unit admission following elective pediatric upper airway surgery.

**Methods:**

We performed a retrospective case–control study at a single tertiary care center, identifying all unplanned pediatric intensive care unit admissions between January 2017 and June 2022. Among 151 such admissions, 29 followed elective upper airway surgery; of these, 22 cases were successfully matched on procedure type, age, and date of surgery in a 1:2 ratio to controls without unplanned pediatric intensive care unit admission based on procedure type, age, and date of surgery. Demographic, clinical, and perioperative variables were compared between cases and controls.

**Results:**

The unplanned pediatric intensive care unit admission (*n* = 22) and control (*n* = 44) groups were comparable with respect to age and weight; however, median apnea‐hypopnea index was higher in the unplanned pediatric intensive care unit cohort (12.7 [IQR 7.9–33.7] vs. 6.6 [IQR 3.7–25.2] events/h; *p* = 0.033). In unadjusted conditional logistic regression analyses, American Society of Anesthesiologists physical status > 2 was associated with increased odds of unplanned pediatric intensive care unit admission (OR 7.92, 95% CI 2.25–27.92; *p* = 0.001), as were prior neonatal intensive care unit admission (OR 23.19, 95% CI 3.03–177.38; *p* = 0.003), chronic lung disease (OR 14.00, 95% CI 1.72–113.79; *p* = 0.014), and longer operative duration (OR 1.03 per minute, 95% CI 1.003–1.058; *p* = 0.032). Apnea‐hypopnea index, analyzed using multiple imputed values, was not significant (OR 1.03 per unit increase, 95% CI 0.996–1.069; *p* = 0.082). In a multivariable conditional logistic regression model, only prior neonatal intensive care unit admission remained independently associated with unplanned pediatric intensive care unit admission (adjusted OR 14.65, 95% CI 1.23–175.05; *p* = 0.034).

**Conclusion:**

Several clinical factors were seen to be associated with increased risk of unplanned pediatric intensive care unit admission after upper airway surgery including American Society of Anesthesiologists physical status > 2, chronic lung disease, prior neonatal intensive care unit admission, and longer operative duration. Controlling for these clinical factors, prior neonatal intensive care unit admission best predicted the need for unplanned pediatric intensive care unit admission. Recognition of these risk factors may help inform perioperative risk stratification and postoperative resource planning.

## 1 Introduction

The Pediatric Intensive Care Unit (PICU) plays a crucial role in caring for critically ill children with a wide range of acute medical conditions that require intensive monitoring and organ support. In addition to medical illnesses, the PICU routinely provides postoperative care for children undergoing complex surgical procedures who are at increased risk for respiratory, hemodynamic, or neurologic complications [1]. Because many elective pediatric surgeries are anticipated to require postoperative critical care monitoring, PICU admission is often planned in advance to ensure appropriate staffing, resource availability, and a safe transition of care from the operating room [2]. Planned postoperative PICU admissions enable proactive risk mitigation and coordinated perioperative management for medically complex pediatric patients.

In some cases, patients require an unplanned postoperative PICU (U‐PICU) admission. General U‐PICU admissions have been shown to contribute to patient morbidity and mortality and are a patient safety concern [3, 4]. In addition, many U‐PICU admissions involve patients who ultimately require only close observation rather than true PICU‐level interventions, contributing to inefficient resource utilization [4, 5, 6, 7]. In healthcare settings where capacity is limited, these unplanned admissions further strain bed availability and staffing resources [7, 8]. Collectively, U‐PICU admissions impose a substantial economic burden on both healthcare systems and patients [8].

Prior studies have identified multiple patient‐ and procedure‐level factors associated with postoperative U‐PICU admission, with airway‐related and anesthetic complications accounting for a substantial proportion of cases [9]. Upper airway surgeries, such as tonsillectomy, adenoidectomy, and cleft palate repair, are among the most commonly performed pediatric procedures and are frequently associated with postoperative respiratory events [10]. Although factors such as higher American Society of Anesthesiologists (ASA) physical status, longer operative duration, and underlying respiratory or neuromuscular comorbidities have been linked to increased risk of U‐PICU admission, existing studies often aggregate heterogeneous surgical populations or focus on specific diagnoses rather than perioperative risk stratification within elective upper airway procedures [6, 9, 11]. As a result, predictors of U‐PICU admission in this high‐volume surgical group remain incompletely defined.

To address this gap, the primary objective of this study was to identify risk factors associated with U‐PICU admissions following upper‐airway surgery. We hypothesized that greater perioperative complexity (Operative time) and underlying medical vulnerability (ASA > 2, prior NICU stay, chronic lung disease) would be associated with increased odds of U‐PICU admission following elective upper airway procedures.

## 2 Methods

### 2.1 Study Design

This was a retrospective case–control study conducted at a single tertiary care institution. Pediatric surgical records spanning a five‐year period (January 2017–June 2022) were queried to identify all U‐PICU admissions occurring within 24 h of surgery. The study was approved by the Pennsylvania State Institutional Review Board (STUDY00017935). The requirement for informed consent was waived due to the study's retrospective nature and the use of de‐identified patient data. All methods were conducted in accordance with institutional policies and the ethical standards outlined in the Declaration of Helsinki. This manuscript was prepared in accordance with the STROBE guidelines for reporting observational studies [12].

### 2.2 Selection of Participants

Pediatric patients with U‐PICU admission following elective upper airway surgery were identified through routine institutional quality assurance reporting. Cases included patients admitted directly from the operating room to the PICU, identified by the Pediatric Anesthesiology Quality Coordinator, and patients upgraded to the PICU within 48 h postoperatively, identified by the Children's Surgical Center and Perinatal Program. These individuals were identified as part of standard clinical and administrative workflows, and the involved staff had no role beyond routine data identification and were not included as study personnel. Case identification was cross‐validated using the Virtual PICU Systems LLC database to ensure completeness and accuracy.

Inclusion criteria were age under 18 years, outpatient status prior to the procedure, elective surgical cases, and U‐PICU admission postoperatively. Electronic medical records were reviewed to identify children undergoing upper airway procedures, including tonsillectomy and/or adenoidectomy (T&A), cleft palate repair, direct rigid laryngoscopy/bronchoscopy performed by otolaryngology, and tracheostomy‐related procedures in patients with a history of tracheostomy.

Controls were selected from upper airway cases that did not have adverse events during the same period. Two controls were matched to each U‐PICU case by procedure type, age (±1 year), and date of procedure (±1 year). Due to challenges in obtaining matched controls for rigid laryngoscopy/bronchoscopy and tracheostomy site procedures (7 cases), the case–control analysis was limited to patients undergoing T&A and cleft palate repair.

The nature of this study, as retrospective in design, precluded a sample size calculation. The sample size was determined by the number of eligible patients who met study criteria during the study period. All available matched cases of unplanned PICU admission and corresponding controls were included in the analysis. While the modest sample size may limit statistical power, a case–control design and use of conditional logistic regression were intended to maximize the efficiency of the available data.

### 2.3 Variables

Data collected included demographic information, relevant medical history, perioperative details (including operative time, airway management, and administered medications), and postoperative outcomes, such as airway interventions and indications for U‐PICU admission. Preoperative and perioperative variables analyzed included documented history of reactive airway disease, chronic lung disease or lung disease of prematurity, history and severity of obstructive sleep apnea, neonatal intensive care unit (NICU) stay > 48 h, tracheostomy dependence at the time of procedure, operative duration, intraoperative dexamethasone dose, and total intraoperative and immediate postoperative doses of intravenous fentanyl and morphine. The immediate postoperative period was defined as the patient's stay in phase I of the post‐anesthesia care unit (PACU).

Intraoperative medication administration was analyzed using clinically relevant dosing thresholds. Dexamethasone administration was considered clinically significant if the dose exceeded 4 mg or 300 mcg/kg in lower‐weight patients. This is the guideline‐recommended dose for perioperative antiemetic prophylaxis, with no additional benefit from higher doses [13]. Opioid administration thresholds were defined as fentanyl > 2 mcg/kg, morphine > 50 mcg/kg, or total opioid exposure exceeding 250 mcg/kg morphine milligram equivalents (MME). These opioid doses were chosen as doses associated with an increased risk of respiratory depression [14]. Severe obstructive sleep apnea was defined as an apnea‐hypopnea index (AHI) ≥ 30 events per hour based on polysomnography, which is the established standard threshold [15]. Chronic lung disease was defined as a documented history of lung disease of prematurity or bronchopulmonary dysplasia. These diseases were chosen because existing guidelines recognize that prematurity‐associated respiratory diseases lead to lifelong respiratory sequelae and increased perioperative vulnerability [16].

### 2.4 Analysis

Baseline characteristics of U‐PICU and non‐PICU patients were compared using descriptive statistics. Continuous variables were assessed for normality, with normal distributions presented as mean ± standard deviation, and were compared using two‐tailed Student's *t*‐tests. Non‐normal distributions were presented as medians with interquartile ranges (IQRs) and were compared using the Mann–Whitney U test. If variables were categorical, they were reported as counts and percentages and compared using chi‐square or Fisher's exact test, depending on sample size.

The continuous variables analyzed in this study included age (months), operative time (minutes), body mass index (BMI), and the apnea‐hypopnea index (AHI). Due to the matched nature of this study, associations were evaluated using conditional logistic regression. Unadjusted conditional logistic regression models were first used to assess each explanatory variable individually. Select variables that demonstrated a *p* < 0.10 in unadjusted analyses were subsequently considered for inclusion in a multivariable model.

Missing AHI values were addressed using multiple imputation, with estimates pooled across 10 imputed datasets. For the case–control analysis, procedural type, age, and date of surgery were matched between cases and controls to minimize confounding.

## 3 Results

### 3.1 Demographics

**FIGURE 1:**
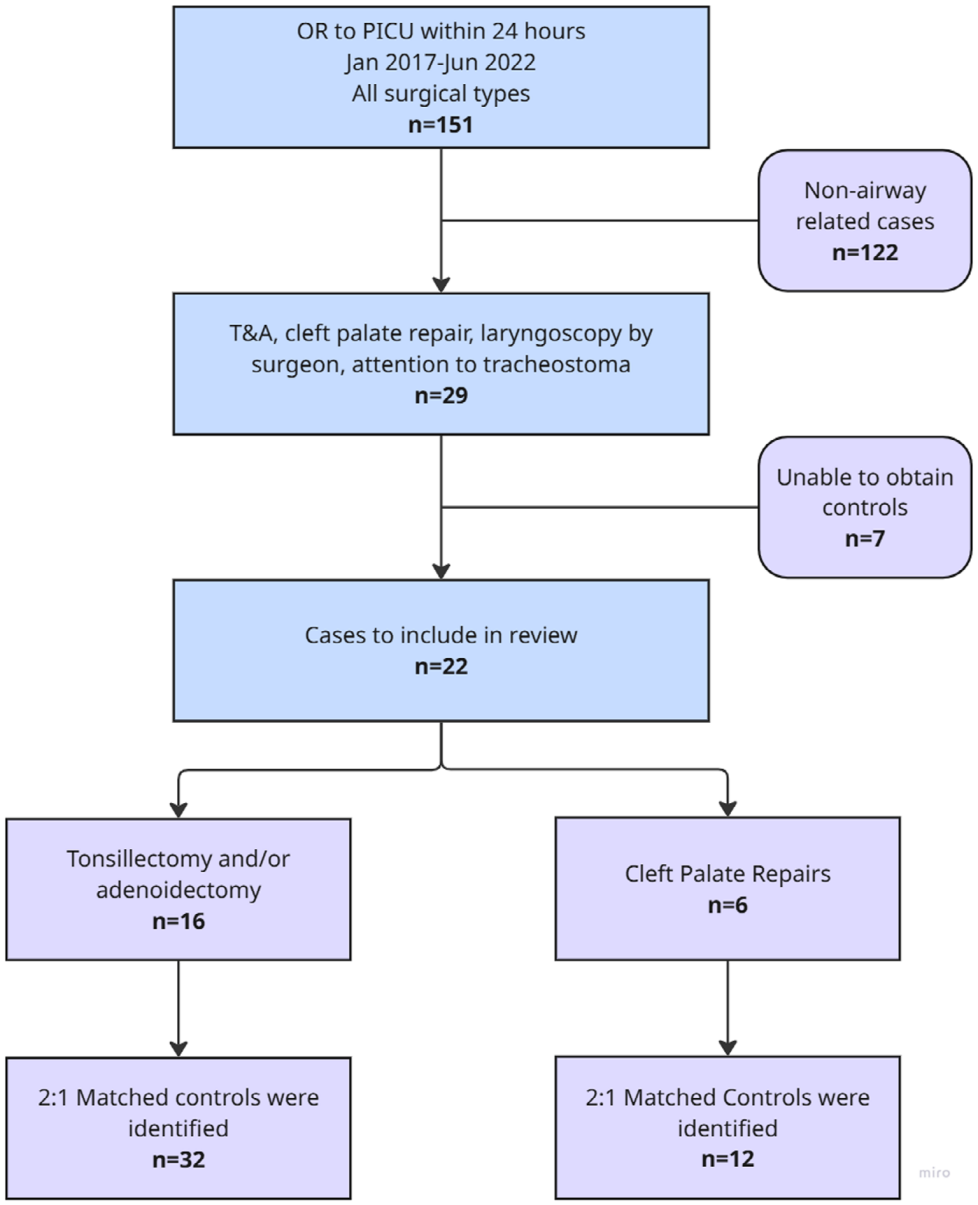
Inclusion/Exclusion Flowchart.

A total of 153 unique patient‐procedure pairs met the inclusion criteria. Of these, 29 cases (19%) involved elective upper airway procedures, including 16 (10.5%) tonsillectomy and adenoidectomy (T&A), 6 (3.9%) cleft palate repairs, 4 (2.6%) combined rigid laryngoscopy/bronchoscopy procedures, and 3 (2%) tracheostomy site‐related procedures. Due to challenges in obtaining matched controls for rigid laryngoscopy/bronchoscopy and tracheostomy site‐related procedures (7 cases total), subsequent case–control analyses were limited to patients undergoing T&A and cleft palate repair. This resulted in 22 U‐PICU admissions (16 T&A, 6 cleft palate repairs) matched to 44 non‐PICU controls (32 T&A, 12 cleft palate repairs), all performed at a single institution. The cohort selection and matching process are illustrated in Figure 1.

### 3.2 Patient Characteristics

Baseline demographic and clinical characteristics of the U‐PICU and non‐PICU cohorts are summarized in Table 1. There were no significant differences in sex distribution between cohorts. Median age did not differ significantly between the U‐PICU and non‐PICU groups (24.5 months [IQR 19–34] vs. 28.5 months [IQR 24–33], *p* = 0.34). Median body mass index was similar between the U‐PICU cohort and the non‐PICU cohort (17.8 [IQR 15.5–19.2] vs. 17.3 [IQR 16.1–18.2], *p* = 0.94). No difference in race or gender was observed (all Fisher's exact *p* > 0.05).

**TABLE 1:** Baseline demographic and clinical characteristics of patients with and without unplanned pediatric intensive care unit admission following elective pediatric upper airway surgery.

Variable	U‐PICU (*n* = 22)	Non‐PICU (*n* = 44)	*p*
Age, months, median (IQR)	24.5 (19–34)	28.5 (24–33)	0.34*
Female sex, *n* (%)	9 (40.9%)	20 (45.5%)	0.79
Race, *n* (%)			0.11
White	10 (45.5%)	31 (70.5%)	
Black	2 (9.1%)	2 (4.5%)	
Asian	1 (4.5%)	2 (4.5%)	
Other/declined	9 (40.9%)	9 (20.5%)	
Apnea‐hypopnea index, events/h, median (IQR)	12.7 (7.9–33.7)	6.6 (3.7–25.2)	0.033*
BMI, median (IQR)	17.8 (15.5–19.2)	17.3 (16.1–18.2)	0.94*
Operative time, minutes, median (IQR)	32.5 (17.8–124.3)	20.0 (16.5–51.5)	0.21*
Surgery type, *n* (%)			1
Tonsillectomy ± adenoidectomy	16 (72.7%)	32 (72.7%)	
Cleft palate repair	6 (27.3%)	12 (27.3%)	

*Note:* Mann–Whitney U test used for continuous variables. Categorical variables were compared using chi‐square or Fisher's exact test as appropriate.

Abbreviations: BMI, body mass index; IQR, interquartile range; PICU, pediatric intensive care unit; U‐PICU, unplanned pediatric intensive care unit admission.

* Apnea‐hypopnea index data was available in 13 of 16 unplanned PICU patients and 42 of 44 controls. Values are presented as medians (interquartile ranges) for continuous variables and counts (percentages) for categorical variables.

Among patients undergoing tonsillectomy and adenoidectomy, the U‐PICU cohort demonstrated a significantly higher AHI compared with the non‐PICU cohort, consistent with more severe obstructive sleep‐disordered breathing. Median AHI was 12.7 events/h (IQR 7.9–33.7) in the U‐PICU group compared with 6.6 events/h (IQR 3.7–25.2) in the non‐PICU group (*p* = 0.033). Of the 16 patients undergoing tonsillectomy and adenoidectomy who were admitted to the U‐PICU, 13 (81.3%) had preoperative polysomnography available for review. All patients admitted to the PICU carried a preoperative diagnosis of obstructive sleep apnea or sleep‐disordered breathing.

### 3.3 Unadjusted Conditional Logistic Regression

Upon unadjusted conditional logistic regression analysis, we identified several variables that predicted U‐PICU admission. Younger patient age was associated with increased odds of U‐PICU admission (OR 0.85 per month increase, 95% CI 0.72–0.996, *p* = 0.045), as were ASA physical status > 2 (OR 7.92, 95% CI 2.25–27.92, *p* = 0.001), history of chronic lung disease (OR 14.0, 95% CI 1.72–113.79, *p* = 0.014), prior NICU admission > 48 h (OR 23.19, 95% CI 3.03–177.38, *p* = 0.003), severe obstructive sleep apnea (OR 3.48, 95% CI 1.17–10.34, *p* = 0.025), and longer operative time (OR 1.03 per unit increase, 95% CI 1.003–1.058, *p* = 0.032).

Several variables were not associated with U‐PICU admission. These included morphine administration (OR 0.29, 95% CI 0.079–1.07, *p* = 0.064), asthma (OR 1.94, *p* = 0.247), dexamethasone administration (OR 1.82, *p* = 0.376), female sex (OR 0.82, *p* = 0.715), and fentanyl administration (OR 0.22, *p* = 0.154).

The analysis of apnea‐hypopnea index (AHI) was completed with imputed values where missingness was assumed to be missing at random, meaning missingness is explainable using variables already in the dataset. Under this analysis, AHI was not associated with U‐PICU admission (OR 1.03, 95% CI 0.996–1.069, *p* = 0.082). This is demonstrated in Table 2.

**TABLE 2:** Results of the unadjusted conditional logistic regression.

Variable	Odds ratio (OR)	95% CI	*p*
Age (per month increase)	0.85	0.72–1.00	0.045
AHI (imputed)	1.03	1.00–1.07	0.082
ASA > 2	7.92	2.25–27.92	0.001
Asthma	1.94	0.63–5.92	0.247
Dexamethasone administration	1.82	0.48–6.89	0.376
Female sex	0.82	0.28–2.43	0.715
Fentanyl administration	0.22	0.03–1.75	0.154
Chronic lung disease	14	1.72–113.79	0.014
Morphine administration	0.29	0.08–1.07	0.064
Prior NICU admission	23.19	3.03–177.38	0.003
Operative time	1.03	1.003–1.058	0.032
Severe OSA	3.48	1.17–10.34	0.025

Abbreviations: AHI, Apnea–Hypopnea Index; ASA, American Society of Anesthesiologists physical status classification; NICU, Neonatal Intensive Care Unit; OSA, obstructive sleep apnea; PICU, Pediatric Intensive Care Unit.

### 3.4 Multivariable Conditional Logistic Regression

Variables that demonstrated a *p* < 0.10 in the unadjusted analyses were eligible for inclusion in multivariable modeling. The relatively small number of outcome events ensured that the inclusion of all eligible variables led to model instability and failure to converge. To address the implied risk of overfitting, a multivariable conditional logistic regression model was restricted to AHI, ASA > 2, chronic lung disease, prior NICU admission, and operative time. This approach prioritized variables representing unique domains of patient risk while ensuring a statistically appropriate events‐to‐variable ratio.

In this multivariable model, only prior NICU admission retained its significantly positive association with U‐PICU admission (OR 14.65, 95% CI 1.23–175.05, *p* = 0.034). The other explanatory variables of AHI (OR 1.05, 95% CI 0.98–1.13, *p* = 0.193), ASA physical status > 2 (OR 3.85, 95% CI 0.34–43.42, *p* = 0.275), chronic lung disease (OR 11.64, 95% CI 0.34–396.20, *p* = 0.173), and operative time (OR 1.04, 95% CI 0.98–1.09, *p* = 0.179) did not retain significant associations with PICU admission after adjustment. This is demonstrated in Table 3.

**TABLE 3:** Multivariable conditional logistic regression analysis of predictors of unplanned pediatric intensive care unit admission.

Variable	Adjusted odds ratio	95% CI	*p*
AHI (imputed)	1.05	0.98–1.13	0.193
ASA > 2	3.85	0.34–43.42	0.275
Chronic lung disease	11.64	0.34–396.20	0.173
Prior NICU admission	14.65	1.23–175.05	0.034
Operative time	1.04	0.98–1.09	0.179

Abbreviations: AHI, apnea‐hypopnea index; ASA, american society of anesthesiologists physical status classification; CI, confidence interval; NICU, neonatal intensive care unit.

### 3.5 Indications and Timing of U‐PICU Admission

When analyzing the indications for U‐PICU admission in these 22 cases, the majority were respiratory complications: six patients (27.3%) required only increased oxygen support, four patients (18.2%) required noninvasive positive pressure ventilation, and 12 patients (54.5%) required mechanical ventilation. Looking specifically at the 12 intubated cases, seven (31.8%) remained tracheally intubated or were reintubated in the operating room, and five (22.7%) required tracheal reintubation in the PICU. These cases of tracheal re‐intubation in the PICU included three cases of hypoxic respiratory failure, one case of apnea, and one case of stridor with concern for airway compromise. 1 patient experienced cardiac arrest on induction. No patients were admitted solely for hemodynamic management, although 1 mechanically ventilated patient also required PICU care for hemodynamic instability. The average hospital length of stay for U‐PICU patients was 8.59 days, with a mean PICU stay of 5.83 days.

Timing of U‐PICU upgrade varied across the perioperative setting. 11 patients (50%) were upgraded from PACU phase I, 11 patients (50%) from PACU phase 2 or the floor status, and 7 patients (31.8%) directly from the operating room. This analysis provides a descriptive overview of the locations from which patients required escalation to intensive care within the subset used for case–control comparisons.

## 4 Discussion

### 4.1 Key Results

This retrospective case–control analysis of elective pediatric upper airway surgery revealed that several patient and procedure‐level factors were positively associated with postoperative U‐PICU admission. The unadjusted analysis showed that younger age, ASA physical status > 2, history of prolonged NICU stay (> 48 h), longer operative duration, severe OSA, and higher AHI were associated with an elevated risk of U‐PICU admission. Following multivariable model analysis of five clinically relevant variables, prior NICU admission emerged as the only independent predictor of U‐PICU admission. This suggests substantial overlap among markers of medical complexity. These findings suggest a set of procedural and patient‐level factors associated with U‐PICU admission, with prior NICU stay > 48 h as a particularly important indicator of postoperative risk.

### 4.2 Interpretation

U‐PICU admission after elective pediatric upper airway surgery represents an important measure of postoperative risk and resource utilization. These admissions likely reflect a convergence of underlying patient vulnerability and perioperative physiologic stress, particularly related to immature airway control, limited respiratory reserve, and the physiologic demands of surgery and anesthesia [6]. Younger children have less mature ventilatory control, smaller functional residual capacity, and increased susceptibility to airway obstruction and hypoventilation, making them particularly vulnerable in the postoperative period [17]. Similarly, longer operative duration may serve as a surrogate for procedural complexity, prolonged anesthetic exposure, or intraoperative challenges that increase postoperative respiratory risk [11]. Our findings align with existing literature demonstrating that patient vulnerability and procedural complexity are key determinants of U‐PICU admission following pediatric surgery [18].

When examining the indications for escalation of care, respiratory pathology emerged as the dominant driver of U‐PICU admission in our cohort. This pattern mirrors broader pediatric critical care trends, as respiratory conditions have become the leading cause of pediatric ICU admissions over the past two decades [19]. Indications such as invasive or noninvasive ventilatory support, impending respiratory failure, or significant oxygen requirements are well‐established triggers for ICU‐level care [20]. Our findings suggest that postoperative respiratory instability, rather than hemodynamic or neurologic complications, is the primary mechanism driving U‐PICU admission following elective upper airway surgery.

Several variables associated with U‐PICU admission in the unadjusted analyses were no longer statistically significant in the multivariable model. This likely reflects substantial overlap among markers of pediatric medical complexity rather than a lack of clinical relevance. Variables such as higher ASA physical status, chronic lung disease, and increased OSA severity each capture related aspects of underlying physiologic vulnerability, particularly impaired respiratory reserve and increased susceptibility to perioperative airway compromise [6, 21]. When these factors were evaluated simultaneously, prior NICU admission appeared to account for a large proportion of the shared variance, suggesting that it serves as a composite marker of early‐life respiratory and medical fragility. In this context, the attenuation of other predictors in the adjusted model likely reflects the clustering of comorbid conditions commonly seen in children with complex neonatal histories [22].

These findings have important implications for perioperative risk assessment in pediatric airway surgery. Identification of prior NICU admission as a strong predictor of postoperative PICU admission may help clinicians refine preoperative risk stratification and guide postoperative monitoring decisions. Children with a history of NICU admission may represent a population with reduced respiratory resilience and a lower threshold for postoperative respiratory decompensation, even when undergoing elective procedures [23]. Recognition of this risk profile may support heightened perioperative vigilance, including extended postoperative monitoring, cautious titration of respiratory depressant medications, and a lower threshold for planned ICU observation in select patients. Incorporating neonatal history into perioperative risk assessment may therefore provide a practical and readily identifiable marker to improve postoperative safety in pediatric airway surgery.

### 4.3 Limitations

This study has several important limitations. First, it represents a single‐center retrospective quality improvement analysis focused on a narrow subset of pediatric upper airway procedures, which limits the generalizability of the findings to other institutions, surgical populations, and practice environments. The number of U‐PICU admissions eligible for inclusion was relatively small, thereby limiting statistical power and increasing the risk of both type I and type II errors. As a result, some clinically meaningful associations may not have been detected, and the stability of effect estimates, particularly in regression analyses, may be limited. In addition, the modest sample size constrained our ability to adjust for multiple potential confounders simultaneously and to evaluate less prevalent risk factors, such as uncommon comorbid conditions or infrequent perioperative exposures.

In addition, this study has limitations relating to the difficulty in obtaining matched controls for 7 cases. In our initial search, we identified 29 postoperative UPICU admissions after upper airway surgery; 22 of these included tonsillectomy and/or adenoidectomy and cleft palate repair, and the remaining 7 included four direct laryngoscopy or rigid bronchoscopy procedures performed by the surgeon and three tracheostomy site procedures. Because these cases are booked through our surgical scheduling system (under the headers “endoscopy” or “tracheostomy”), it was difficult to identify specific controls for these procedures. Additionally, all elective tracheostomy procedures are planned with PICU status at our institution, and there were no outpatient procedures under the scheduling header “tracheostomy” in the random controls. This introduces a potential selection bias into this study due to unmatched cases; however, these cases.

Given that the study period from 2017 to 2022 included the COVID19 pandemic, it may complicate the interpretation of the findings. Perioperative management strategies, anesthetic techniques, postoperative monitoring protocols, and institutional thresholds for PICU admission likely evolved over this interval, introducing temporal heterogeneity that our analyses could not fully account for. The retrospective design and relatively short timeframe also limited the number of eligible cases available for this study, particularly for less common procedures, and precluded standardized assessment of some variables. Finally, reliance on electronic medical record data introduces the potential for misclassification, incomplete documentation, and residual confounding despite the matching of cases and controls.

Taken together, these limitations highlight the need for prospective, multi‐institutional studies with larger sample sizes and contemporary practice patterns to validate these findings and refine perioperative risk stratification for children undergoing elective upper airway surgery.

## 5 Conclusion

In this retrospective case–control study of pediatric patients undergoing elective upper airway surgery, multiple markers of medical and respiratory vulnerability were associated with increased risk of unplanned PICU admission. These included American Society of Anesthesiologists physical status > 2, chronic lung disease, prior neonatal intensive care unit admission, and longer operative duration. Upon controlling for these clinical factors, prior NICU admission remained positively associated with postoperative PICU admission, suggesting that neonatal history may serve as a composite marker of persistent respiratory vulnerability. These findings highlight the importance of considering early‐life medical history in perioperative risk stratification for pediatric airway surgery. Incorporation of neonatal history into preoperative assessment may help identify patients who would benefit from enhanced postoperative monitoring and from targeted perioperative management strategies.

## Author Contributions

**Hayes Stancliff:** statistical analysis, drafting of the manuscript, and critical revision of the manuscript. **Michelle M. Basilious:** study conceptualization, statistical analysis, and critical revision of the manuscript. **Lisa R. Yoder:** study conceptualization, statistical analysis. **Conrad Krawiec:** critical revision of the manuscript, expert opinion on study design and conceptualization. **Terrence Murphy:** statistical Analysis, Critical revision of the manuscript. **Priti G. Dalal:** critical revision of the manuscript, expert opinion on study design and conceptualization.

## Funding

This work was supported by the National Center for Advancing Translational Sciences, UL1 TR002014.

## Ethics Statement

This study was approved by the Penn State Institutional Review Board (STUDY00017935). The requirement for informed consent was waived due to the retrospective nature of the study and the use of de‐identified patient data. All methods were conducted in accordance with institutional policies and the ethical standards outlined in the Declaration of Helsinki.

## Consent

The requirement for informed consent was waived due to the retrospective nature of the study and the use of de‐identified patient data.

## Conflicts of Interest

The authors declare no conflicts of interest.

## Data Availability

The data that support the findings of this study are available from the corresponding author upon reasonable request.
